# Insights into Potential Targets for Therapeutic Intervention in Epilepsy

**DOI:** 10.3390/ijms21228573

**Published:** 2020-11-13

**Authors:** Cecilia Zavala-Tecuapetla, Manola Cuellar-Herrera, Hiram Luna-Munguia

**Affiliations:** 1 Laboratory of Physiology of Reticular Formation, National Institute of Neurology and Neurosurgery-MVS, Insurgentes Sur 3877, La Fama, 14269 Mexico City, Mexico; 2 Epilepsy Clinic, Hospital General de México Dr. Eduardo Liceaga. Dr. Balmis 148, Doctores, 06720 Mexico City, Mexico; manolacuellar@yahoo.com.mx; 3 Departamento de Neurobiologia Conductual y Cognitiva, Instituto de Neurobiologia, Campus UNAM-Juriquilla, Universidad Nacional Autonoma de Mexico, 76230 Queretaro, Mexico; hiram_luna@inb.unam.mx

**Keywords:** epilepsy, seizures, drug-resistant epilepsy, antiseizure efficacy, antiepileptogenic effect, neuroprotective effect

## Abstract

Epilepsy is a chronic brain disease that affects approximately 65 million people worldwide. However, despite the continuous development of antiepileptic drugs, over 30% patients with epilepsy progress to drug-resistant epilepsy. For this reason, it is a high priority objective in preclinical research to find novel therapeutic targets and to develop effective drugs that prevent or reverse the molecular mechanisms underlying epilepsy progression. Among these potential therapeutic targets, we highlight currently available information involving signaling pathways (Wnt/β-catenin, Mammalian Target of Rapamycin (mTOR) signaling and zinc signaling), enzymes (carbonic anhydrase), proteins (erythropoietin, copine 6 and complement system), channels (Transient Receptor Potential Vanilloid Type 1 (TRPV1) channel) and receptors (galanin and melatonin receptors). All of them have demonstrated a certain degree of efficacy not only in controlling seizures but also in displaying neuroprotective activity and in modifying the progression of epilepsy. Although some research with these specific targets has been done in relation with epilepsy, they have not been fully explored as potential therapeutic targets that could help address the unsolved issue of drug-resistant epilepsy and develop new antiseizure therapies for the treatment of epilepsy.

## 1. Introduction

Epilepsy is a chronic, recurrent and progressive neurological disease, which affects approximately 65 million people worldwide [1]. It is characterized by recurrent spontaneous epileptic seizures that result from abnormal and excessive electrical discharges. Despite many advances in epilepsy research, approximately 30% of all patients show drug-resistant epilepsy [2], which is defined as the persistence of epileptic seizures despite the application of an adequate and well-tolerated pharmacological treatment [3]. Temporal lobe epilepsy (TLE) is the most common form of epilepsy and often resistant to antiepileptic drugs (AEDs) [4,5].

The pathophysiological mechanisms that underlie the development of chronic epilepsy, or epileptogenesis, are still unclear [6]. The progression of the disease most likely involves a combination of neurological changes in the brain [7]. Finding novel therapeutic targets and developing effective drugs that prevent or reverse the molecular mechanisms underlying epilepsy progression are priority objectives in preclinical research.

This review describes in detail the recent advances in the search for potential therapeutic targets for the treatment of epilepsy, with a special emphasis on the variety of signaling pathways (Wnt/β-catenin, mTOR and zinc signaling), enzymes (carbonic anhydrase), proteins (erythropoietin, copine 6 and complement system), channels (TRPV1 channel) and receptors (galanin and melatonin receptors) involved in the development and progression of the disease. We give the prospects of their application as a treatment not only for seizures or epilepsy but also for drug-resistant epilepsy.

Based on preclinical and clinical evidence, all of these targets have demonstrated a certain degree of efficacy not only in controlling seizures but also in displaying neuroprotective activity (protecting brain areas against seizure-related brain damage) and in modifying the progression of epilepsy (antiepileptogenic intervention). In this review, we choose these specific targets because, while some research with them has been done in relation with epilepsy, they have not been fully explored as potential therapeutic drugs that could help address the unsolved issue of drug-resistant epilepsy and develop more efficient drugs for the treatment of epilepsy.

## 2. Wnt Signaling Pathway

Since its discovery, the so-called *Wingless/integrase-1 (Wnt) gene* and its downstream signaling, have been related to fundamental biological processes, including embryonic development, neurogenesis, synaptogenesis and adult neural plasticity [8,9].

Three different branches of signaling pathways mediated by Wnt proteins have been identified: (a) the canonical pathway, also called the Wnt/β-catenin pathway, involves the stabilization of the proto-oncogene β-catenin [10,11,12]; (b) noncanonical (β-catenin-independent) Wnt signaling or planar cell polarity (PCP) pathways, which are required to establish tissue polarity of many epithelia [12,13,14]; and (c) the Wnt/Ca^2+^ pathway, which stimulates the intracellular release of Ca^2+^ and activates calcium-dependent mediators [12,15,16].

The Wnt/β-catenin signaling pathway is the most characterized signaling cascade and its activation has been prominently involved in regulating cell differentiation and proliferation.

### 2.1. Wnt/β-Catenin Pathway

**Figure 1:**
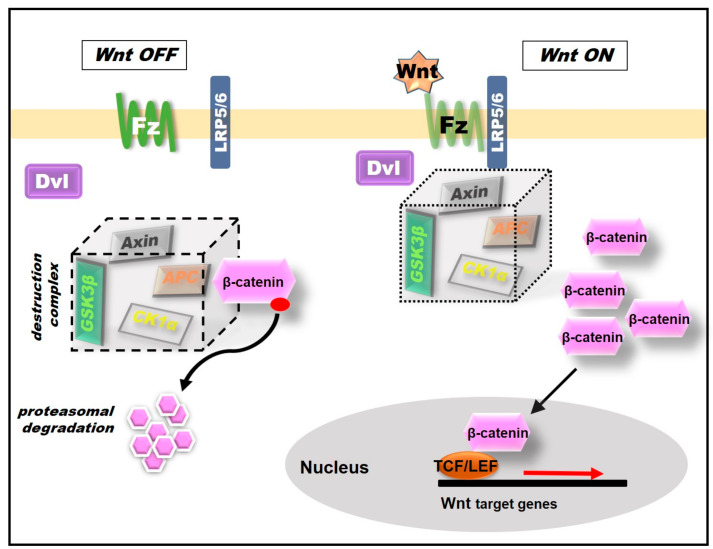
Overview of Wnt/β-catenin pathway. In the absence of Wnt stimulation (Wnt OFF), cytoplasmic levels of β-catenin are low since it is phosphorylated by the destruction complex, resulting in recognition and proteasomal degradation. Once the Wnt-Fz-LRP5/6 interaction (Wnt ON) has started, the destruction complex is disassembled and therefore the proteasomal degradation of β-catenin is prevented. In this way, β-catenin accumulates within the cytoplasm and translocates to the nucleus, where it binds to the transcription factors (T-cell factor and lymphoid enhancer factor (TCF/LEF)) to finally activate the transcription of Wnt target genes. LRP5/6, lipoprotein receptor-related protein 5 or 6; Fzd, receptor frizzled; Dvl, dishevelled; APC, adenomatous polyposis coli; CK1α, casein kinase 1α; GSK3β, glycogen synthase kinase 3β.

In steady-state or in the absence of Wnt stimulation, cytoplasmic levels of β-catenin are low because of its degradation by a cytoplasmic destruction complex, formed by axis inhibition protein (Axin), Adenomatous Polyposis Coli (APC), constitutively active Casein Kinase-1α (CK-1α) and Glycogen Synthase Kinase 3β (GSK3β) [17,18,19]. β-catenin is phosphorylated by CK-1α and GSK3β, resulting in recognition and ubiquitination, followed by proteasomal degradation [17,20] (Figure 1).

To initiate the Wnt/β-catenin signaling transduction pathway [9,10,12,21], Wnt proteins (lipid-modified, secreted proteins of approximately 400 amino acids) bind to cell surface receptor Frizzled (Fz; a seven-transmembrane receptor that contains a cysteine-rich domain) which forms the Wnt-binding site. In addition to Fz, other proteins act as coreceptors such as the single transmembrane low-density lipoprotein receptor-related protein 5/6 (LRP5/6). Upon Wnt-Fz-LRP5/6 interaction, Axin is recruited to the phosphorylated tail of LRP5/6 by the scaffold protein Dishevelled (Dvl) which disassembles the destruction complex. Dvl inhibits the activity of the enzyme GSK3β, which prevents the phosphorylation of β-catenin and posterior ubiquitination. Under these conditions, β-catenin accumulates within the cytoplasm and translocates to the nucleus, where it will displace the Groucho transcriptional repressor and bind to the transcription factors such as DNA-bound T-cell factor/lymphoid enhancer-binding factor 1 (TCF/LEF) to finally activate the transcription of Wnt target genes (Figure 1). Several Wnt target genes are expressed in this process, including *c-Myc*, *cyclinD1*, *Axin2* and Ca^2+^/calmodulin-dependent protein kinase type IV (CamKIV) [22,23].

### 2.2. Wnt/β-Catenin Pathway and Epilepsy

Several lines of evidence have shown that the Wnt/β-catenin pathway serves as a key bimodal regulator of neurogenesis, facilitating both positive and negative regulation of neuronal homeostasis [24]. In relation to epilepsy, the Wnt/β-catenin pathway has been involved with seizure-induced neurogenesis and neuronal death during the acute and chronic phases of epilepsy.

By different acute seizures models and epilepsy models, elevated expressions of Wnt/β-catenin pathway components have been found, which are associated with the increased neurogenesis and neuronal death commonly observed after seizures [25,26,27,28].

For example, chronic electroconvulsive seizures produced an upregulation of both Wnt2 expression and levels of β-catenin immunoreactivity in the subgranular zone of the adult rat hippocampus, showing that seizure activity regulates components of the Wnt/β-catenin pathway [25].

In another study, employing the amygdaloid kindling model, it was found that rats that received a major number of stimuli (45 stimuli) showed the highest levels of β-catenin in the cerebellum, suggesting the Wnt/β-catenin pathway as one of the mechanisms of generalized seizures generation [26].

Theilhaber et al. [27] investigated how functional pathways could be activated during hypoxic seizures by microarray profiling. They found a gene expression increase of several components of the Wnt/β-catenin pathway including genes for β-catenin (*CTNNB1*), inhibitory protein SFRP2 and transducer Dvl3; co-receptor LRP6; the β-catenin regulatory complex components CSKN1E, GSK3β, Axin2, and APC; and the genes for Frizzled ligands Wnt2, Wnt5a and Wnt10a. Thus, these increases of gene expression may reflect synaptic changes due to the activation of the Wnt/β-catenin pathway, in response to hypoxic seizures.

In an epilepsy model induced by kainic acid (KA), the Wnt/β-catenin pathway was activated by upregulation of the expression levels of its key regulators (Wnt3a, *cyclin D1* and β-catenin) in a time-dependent manner, in the hippocampus of epileptic animals [29].

On the other side, it has been suggested that the Wnt/β-catenin pathway could also have a potential role in regulating seizure susceptibility and epileptogenesis.

Campos et al. [30] employed cre-loxP-mediated conditional mutagenesis to generate mutant mice with restricted deletion of β-catenin (*Catnb conditional knockout mice*, restricted to cerebral cortex and hippocampus). These mice showed, on the one side, missing hippocampal structures as well as cortical dysplasia and, on the other side, increased seizure susceptibility to pentylenetetrazole (PTZ). The above suggests that β-catenin-mediated signaling pathways are critically involved in controlling cortical development, seizure susceptibility and epileptogenesis.

Another study examined microRNAs (miRNA) expression/regulation of signaling pathways involved in epileptogenesis, after electrically-induced status epilepticus (SE) in rats, in a model of TLE [31]. It was found that, after the SE treatment, both significant upregulation and downregulation occurred of the miRNAs that target genes of the Wnt pathway [31], which suggests that silencing or activating specific miRNAs that target Wnt pathway genes could have therapeutic potential.

On the other hand, Yang et al. [28] investigated whether Wnt/β-catenin pathway is involved in the seizure-facilitating effects of reactive astrocytes after ischemia. They found that cerebral ischemia enhanced seizure susceptibility in the PTZ-kindling model and that reactive astrocytes are involved in this ischemia-increased seizure susceptibility [28]. More important is that the Wnt/β-catenin pathway was activated in Nestin-positive reactive astrocytes after ischemia and that depletion of β-catenin in reactive astrocytes could attenuate the ischemia-increased seizure susceptibility after PTZ kindling, indicating the requirement of β-catenin for seizure induction [28]. In general, Yang et al. [28] demonstrated the role of astrocytic Wnt/β-catenin pathway in facilitating seizure-induction after ischemia.

Pirone et al. [32] developed conditional knockout mice of the APC protein (*APCcKO*), generating an animal with high levels of β-catenin. These animals showed many of the signs and symptoms developed in infantile spasms (IS, a common childhood epilepsy syndrome) [33], such as neonatal spasms, abnormal EEG activity in neonates and adults and progression to spontaneous seizures [32]. Excessive β-catenin levels have been associated with aberrant dendritic and axonal branching, increased excitatory synapse density and altered synaptic maturation and function [34,35]. Such changes in vivo would be consistent with neural circuit hyperexcitability and increased seizure susceptibility. All of these previous results strongly support the impact of Wnt/β-catenin signaling in epilepsy [32].

The Wnt/β-catenin pathway also plays an important role in apoptosis, as Wnt pathway activation stimulates β-catenin to directly induce the expression of apoptotic-promoting proteins such as c-Myc and cyclin D [26,36,37]. These findings suggest this pathway as one of the mechanisms by which neurons ultimately die during chronic epileptic seizures, just as it was observed in the cerebellar neurons of rats chronically stimulated with the amygdala kindling [26], where higher activation of the Wnt/β-catenin pathway was associated with the increased presence of apoptotic markers such as c-Myc, cyclin D3, TUNEL and caspase 3 [26].

All these results highlight the importance of Wnt/β-catenin signaling in controlling seizure susceptibility, apoptosis, neurogenesis and potentially epileptogenesis in several animal models of epilepsy. Therefore, the modulation of Wnt/β-catenin pathway could have beneficial effects in protecting against neuronal damage and death following seizures [38].

Secreted glycoprotein Dickkopf-1 (Dkk-1) acts as a selective inhibitor of the Wnt/β-catenin pathway [39,40,41] which could contribute to the neuronal damage associated with TLE [38]. A correlation has been found between the induction of Dkk-1 and neuronal death in rats developing seizures in response to kainate injection, as well as a strong expression of Dkk-1 in the hippocampus of patients with TLE associated with hippocampal sclerosis (a common form of epilepsy associated with drug-resistance) [38], where the induction of Dkk-1 might represent a component of the sequence of events leading to neuronal death [42]. In addition, there was a reduction of β-catenin levels in the hippocampal nuclear fraction of these animals, suggesting that Dkk-1 inhibits the Wnt/β-catenin pathway, by limiting the amount of β-catenin available for nuclear translocation [38] and depriving neurons of the activation of a gene program that is essential for their survival [43]. In this way, antagonizing Dkk-1 was found to limit the extent of neuronal damage associated with TLE independently of its effect on seizure activity [38]. Pretreatment with either Dkk-1 antisense oligonucleotides or lithium ions reduced kainate-induced neuronal damage [38]. Lithium ions are known to rescue the Wnt/β-catenin pathway by inhibiting GSK3β, this is, by acting downstream of the Dkk-1 blockade [44].

For these reasons, Dkk-1 antagonists [38,45,46] or GSK3β inhibitors [38,47,48,49,50,51] could be promising therapeutic candidates for epilepsy. However, it is necessary to delimit the time window to suppress or enhance neurogenesis by the modulation of Wnt/β-catenin signaling, either at the beginning or once epilepsy has fully developed, in order to establish when modulation of the pathway may be the most beneficial as a therapeutic target in epilepsy.

## 3. The Mammalian Target of Rapamycin (mTOR) Signaling Pathway

The mammalian target of rapamycin (mTOR) is a kinase ubiquitously expressed that belongs to the phosphoinositide-3-kinase (PI3K) family [52], regulating cell growth, differentiation, proliferation and metabolism by participating in multiple signaling pathways [53]. Specifically, in the brain, it is involved in functions that can affect neuronal signaling and excitability, as well as axonal and dendritic morphology, neurotransmitter receptor expression and synaptic plasticity [53,54,55].

**Figure 2:**
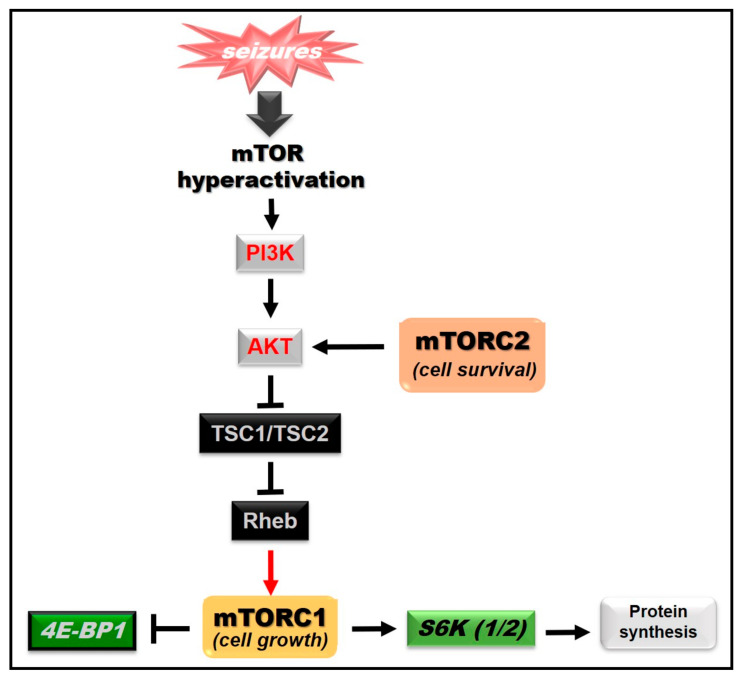
Simplified schematic representation of the hyperactivated mammalian target of rapamycin (mTOR) signaling due to seizures. Once the mTOR pathway is activated, it acts on subsequent effectors to suppress autophagy, as well as promote protein synthesis and cell survival related to the epileptogenesis process. Arrows and bars represent activation and inhibition, respectively. PI3K, phosphatidylinositol 3-kinase; AKT, protein kinase B; TSC1/TSC2, tumor suppressor complex 1/2; Rheb, ras homolog enriched in brain; mTORC1, mTOR complex 1; mTORC2, mTOR complex 2; 4E-BP1, eukaryotic translation initiation factor 4E-binding protein 1; S6K, ribosomal S6 kinase.

In mammalian cells, mTOR is encoded as a single gene whose protein product signals via two different complexes known as mTOR complex 1 (mTORC1) and mTOR complex 2 (mTORC2) [56,57]. Each complex possesses different substrates, cellular functions and sensitivity to rapamycin. In the case of rapamycin-sensitive mTORC1, it suppresses autophagy and its signaling is inhibited by rapamycin and its derivatives (allosteric mTOR inhibitors) [58]. Moreover, it promotes lipid, nucleotide and protein synthesis, as well as cell growth and cell proliferation via activation of S6K (1/2) and inhibition of the translation repressor 4E-BP1 (Figure 2) [53,59]. In fact, the phosphorylation of S6 and S6K1 is considered as a biomarker for mTORC1 activation in neurons [60] and an important contributor to the effects of mTORC1 hyperactivation [61].

On the other hand, rapamycin-insensitive mTORC2 is a key regulator of the actin cytoskeleton and is insensitive to any rapamycin treatment [53,58,59]. In addition, it promotes cell survival via activation of Akt/PKB (protein kinase B) (Figure 2) [62], which inactivates diverse pro-apoptotic factors [63,64].

Much of the available information regarding mTOR signaling in the brain focuses on mTORC1 as a crucial regulator of neuronal excitability since its involvement in the modulation of several processes such as: (1) cerebral cortical development [65,66]; (2) protein synthesis [67,68]; (3) cell differentiation, proliferation, growth, and survival [69,70,71,72]; (4) axonal sprouting, regeneration, and myelination [73,74]; (5) glial functions [75]; (6) dendritic morphogenesis [76]; (7) ionic and receptor channel modulation [68,77,78]; and (8) some forms of neuronal plasticity such as long-term potentiation, long-term depression, learning and memory [79,80,81]. However, changes in mTORC1 activity are known to result in increases in translation, transcription, autophagy, cell signaling, metabolism and altered cytoskeleton dynamics [82,83] that have been correlated with the pathophysiological development/progression of some psychiatric diseases and neurological disorders such as depression [84,85], schizophrenia [86,87], autism [88,89], Parkinson’s disease [90,91], Alzheimer’s disease [92] and epilepsy [93,94], among others.

### mTOR Signaling and Epilepsy

Considering mTOR involvement in cellular functions influencing neuronal excitability, this signaling pathway could participate in the development of epilepsy (Figure 2), as it has been reported in both animal models of epilepsy and in human tissue samples resected from individuals with epilepsy.

Enhanced mTOR signaling in the brain was first identified in resected specimens from patients with tuberous sclerosis complex and focal cortical displasia [60,95], both considered as focal cerebral cortical malformations that usually cause drug-resistant seizures in children [96,97]. Later studies using different animal models of genetic and acquired epilepsies demonstrated that a hyperactivated mTOR seems to play a crucial role in epileptogenesis [98] and that mTOR inhibitors decrease the development of seizures preventing epileptogenesis-related mechanisms [99].

Zeng et al. [100] were the first to suggest that mTOR could be a potential therapeutic target for epilepsy. They used a mouse model of tuberous sclerosis complex and showed that early treatment with rapamycin not only inhibits the activation of mTOR but also delays astrogliosis and hippocampal pyramidal cell disorganization, preventing the development of epilepsy. Moreover, when rapamycin was administered in already epileptic mice, it can also suppress seizures.

Diverse studies have proven an increased mTOR activation in rodent models of TLE where seizures were chemically-induced by pilocarpine [101] or KA [102,103,104]. A similar effect was described in animals with acute PTZ-induced seizures [105] and in electrically-induced epileptic animals [106]. Particularly, Zeng et al. [102] showed a biphasic activation of mTOR that possibly correlates with the observed epileptogenic events: the first one occurring minutes/hours after the SE-induction and the second one 3–7 days after being induced.

Some experimental reports have suggested that a decreased mTORC2-dependent negative regulation of proapoptotic pathways may be responsible for acute seizure-induced neuronal death [107,108]. Recently, Talos et al. [109] showed that mTORC1 and mTORC2 are highly activated in surgically resected samples from drug-resistant TLE patients. In addition, they established for the first time two neuroprotective signaling pathways downstream from mTORC2. Their results suggest that the chronic use of rapamycin or its derivatives may also affect mTORC2 function. In addition, mTORC1 and mTORC2 seem to be activated in chronic rodent models of TLE [110,111].

All previous results support the notion that a hyperactivated mTOR signaling may contribute to epilepsy and that mTOR inhibitors could act as potential antiepileptogenic or antiepileptic treatments [112].

Treatment with rapamycin in models of epilepsy and acute seizure models, has demonstrated significant variability. In some cases, mTOR inhibition has mitigated acute or chronic seizures, and, in other models, the blockade of mTOR by rapamycin had minimal effect on seizures or epilepsy development [102,113,114,115,116], suggesting that the effects of rapamycin might only be restricted to certain experimental models and conditions [98,117]. This is a good reminder that there are both a vast number of downstream mechanisms that may mediate epileptogenesis and overlapping pathological mechanisms across the different existing types of epilepsy.

In clinical settings, mTOR inhibitors are effective and FDA approved for treating subependymal giant cell astrocytomas in patients with tuberous sclerosis complex. Muncy et al. [118] described for the first time the effectiveness of rapamycin in the treatment of a 10-year-old girl with drug-resistant epilepsy, reporting a significant reduction in seizure frequency. Later studies showed that everolimus, a rapamycin analog, was also effective in the treatment of tuberous sclerosis complex-related epilepsy by reducing the seizure frequency and the volume of the subependymal giant cell astrocytomas [119,120,121,122]. However, rapamycin and its derivatives display non-favorable pharmacokinetics with poor oral bioavailability, low blood–brain barrier (BBB) penetration, as well as slow accumulation in the brain [123,124].

Recently, two highly selective inhibitors have been developed: a mTORC1/C2 inhibitor (PQR620), which targets both mTOR complexes (mTORC1 and mTORC2) that shut down the feedback activation of the mTOR pathway, and a dual pan-PI3K/mTOR inhibitor (PQR530), which, in addition to mTORC1 and mTORC2, also inhibits PI3K, counteracting the feedback activation upstream of mTOR [53,125,126,127]. Both inhibitors seem to rapidly penetrate into the brain, reaching brain plasma levels of >1, and they are eliminated with a half-life of only ~5 h [125,126,127]. However, although PQR620 showed dose-dependent antiseizure efficacy [125], both PQR620 and PQR530 lack an antiepileptogenic effect, that is, they did not prevent or modify epilepsy in a post-SE model of TLE [128], in the same way that rapamycin did not show antiepileptogenic effect in this model [104]. In addition, administration of PQR530 inhibitor was associated with adverse effects, particularly loss of body weight [128].

Therefore, alternative pharmacological approaches to selectively modulate the mTOR pathway seem to be preferable for treating epilepsy. Based on the pharmacokinetics advantages and antiseizure efficacy, particularly of PQR620 inhibitor, it would be interesting to research the antiepileptic potential of mTOR inhibitors, both in chronic animal models of epilepsy and in drug-resistance epilepsy.

## 4. Zinc Signaling

Zinc constitutes one of the most abundant metal ions found in the human brain where it participates in neuronal and synaptic functions [129,130], also it can function as a catalytic factor, a structural component and a signaling mediator [129,130], both extracellularly and intracellularly [131,132,133].

Different transporter proteins exist to modulate and to maintain zinc homeostasis. The zinc transporters (ZnTs: subtypes 1–10) decrease cytoplasmic zinc by taking it out of the cell or by moving it into cellular organelles [134]. Of these transporters, the ZnT3 subtype is found in the synaptic vesicle membrane, and its principal function is to store zinc in the vesicles [135,136], while the ZnT1 subtype is found in the plasma membrane, and its function is to reduce intracellular zinc levels [137,138]. In the brain, the ZnT1 transporter is found in the cortex and hippocampus [139], while other subtypes such as ZnT4, ZnT5 and ZnT6 are also found in diverse brain areas [140,141].

The zinc-regulated and iron-regulated transporters’ (ZIP; subtypes 1–14) function is to increase the cytoplasmic levels by up taking zinc from cellular vesicles or from the extracellular space [132,142,143,144].

Metallothioneins (MTs; subtypes I–IV) are high affinity proteins to zinc which function as zinc’s temporary cytosolic store promoting its union to MTs during periods of increased zinc concentration [145]. The main subtype located in neurons, the MT-III protein, has been found in cerebral areas such as amygdala, cerebellum, cortex and hippocampus [146].

In the brain, it has been reported that zinc can induce or prevent neuronal injury depending on the cell type involved and of the zinc levels in those specific cell types [147,148,149,150]. Through the modulation of different receptors, such as the N-methyl-D-aspartate receptor (NMDAR) or the γ-aminobutyric acid A receptor (GABAAR), zinc is able to regulate both excitatory and inhibitory neurotransmission [151,152,153].

Interestingly, zinc can be released into the synapse along with glutamate, from glutamatergic neurons, during neuronal excitation [154,155,156,157,158]. These glutamatergic neurons containing zinc are mainly found in the hippocampus and the amygdala [153,159].

Additionally, zinc can also modulate the activity of several types of ion channels, such as potassium, calcium or sodium channels, and can modulate some membrane receptors, such as α-amino-3-hydroxy-5-methyl-4-isoxazolepropionic acid receptor (AMPAR) and glycine receptors [160,161,162].

Another receptor that interacts with zinc is the zinc-specific receptor/G protein-linked receptor 39 (mZnR/GPR39) [163,164,165]. This receptor is expressed in cerebral areas such as cortex, amygdala or hippocampus [166], and, upon its activation by zinc, it triggers an increase in Ca+2 release and consequently the activation of a series of intracellular signaling pathways [167].

### Zinc, Seizures and Epilepsy

Zinc is necessary for maintaining the balance between neuronal excitation and inhibition, via modulation of a variety of targets. Thus, it is possible that zinc plays an important role in the pathophysiology of seizures and/or epilepsy.

At the human brain, the increased levels of metallothioneins I/II (MT-I/II) in the hippocampus of patients with drug-resistant TLE and TLE associated with tumor or dysplasia, suggest the involvement of MTs in these pathophysiologies [168]. In addition, deficiencies in plasma zinc levels have been associated with human epileptic disorders [169,170,171]. At flurothyl-induced model of recurrent neonatal seizures, there were changes in the expression of MTs and zinc transporters (ZnTs) [172,173,174,175].

On the other hand, *ZnT-3 knockout mice* are more sensitive to seizures caused by KA injection or hyperthermia due to their lack of synaptic zinc ions [176,177], indicating that a deficiency in synaptic zinc ions reduces seizure thresholds. The *mZnR/GPR39 knockout mice* also presents enhanced susceptibility to KA-induced seizures and increased seizure duration [178].

Potassium chloride cotransporter 2 (KCC2) is a neuron-specific chloride extruding transporter, responsible for hyperpolarizing currents mediated by GABAAR activation [179,180]. Loss of KCC2 function increase seizure susceptibility [181,182], and changes in KCC2 have been tightly linked to human epilepsy [183,184,185,186,187,188]. It has been observed that activation of the mZnR/GPR39 receptor leads to the upregulation of KCC2 surface expression and activity [164,189]. In addition, mZnR/GPR39 activation acts as an adaptive homeostatic response to abnormal excitatory activity (e.g., during epileptiform activity) that enhances the inhibitory drive by enhancing KCC2 activity [178,190]. Thus, by regulating neuronal excitability, ZnR/GPR39 activity can limit neuronal firing and consequently may play a role in epilepsy development and progression.

Besides, there are studies suggesting that seizure activity can be moderated via zinc administration [191,192,193]. However, some results are contradictory due to the type of epilepsy, the dose and the route of administration of zinc.

Of the first kind of studies, it is reported that zinc loading delayed kindled seizure induction, while zinc deprivation accelerated kindling [194]. Subsequently, it was found that zinc deficiency causes increased seizure susceptibility, while it is reduced by zinc loading [195,196,197,198,199]. In addition, the administration of zinc chelators such as clioquinol and TPEN increased the incidence of spontaneously generalized tonic-clonic convulsions [199]. Zinc infusion into the hippocampus delayed the development of behavioral seizures in a kindling model of epilepsy [193], while its oral administration reduced seizure duration and increased the latency of seizures induced by PTZ [200]. However, a proconvulsive effect was observed with zinc´s intracerebroventricularly pretreatment which decreased the threshold and increased the severity of seizures induced by PTZ [201].

In addition, it has been proposed that zinc could have a neurotoxic role where excessive zinc could impair the protein degradation pathway and might be a crucial factor mediating neuronal death [202]. In line with this, another study demonstrated that the pretreatment with a high dose of zinc exacerbated pilocarpine-induced seizures, whereas a medium dose of zinc reduced the severity of limbic seizures [203]. In appropriate doses, zinc plays an important role in mitigating seizures by suppressing oxidative stress, apoptotic activity and IL-1β levels, resulting in neuroprotective effects [203].

Furthermore, it has been proposed that activation of KCNQ channels by intracellular zinc may represent a strategy to survive cell excitotoxicity associated with seizures [204].

All of the above indicates the complexity of zinc signaling in epilepsy, with pro- or anticonvulsant effects, and in some cases neuronal neurotoxicity, most likely due to the differing zinc sources and also because zinc modulates a variety of ion channels and receptors. Therefore, further studies are necessary before suggesting zinc supplementation as a therapeutic strategy to treat seizures, epilepsy or drug-resistant epilepsy. In addition, it is necessary to continue to study new targets associated with zinc signaling to investigate if modulating these proteins will produce anticonvulsant or antiepileptogenic effects.

## 5. Carbonic Anhydrase

Carbonic anhydrases or carbonate dehydratases are zinc-containing metalloenzymes present in most living organisms. They play a crucial role in several metabolic pathways and in the pH balance of several body fluids and tissues by catalyzing the reversible hydration of carbon dioxide (CO_2_) and dehydration of bicarbonate (HCO_3_^−^) in a two-step reaction [205,206,207] Equation (1):

(1) $${\mathrm{CO}}_{2\;}+H_{2}O\;\underset{\mathrm{CA}}{↔}{\mathrm{HCO}}_{3^{-}\;}+\;H^{+}$$

Carbonic anhydrases have been categorized into five classes: α (only one found in mammals), β, γ, δ and ε. Of the five different types, the alpha (α) class has received the most attention due to its role in human pathology. To date, the family of α-carbonic anhydrases comprises 16 different isozymes, identified with different catalytic activities, tissue distributions and subcellular localization (I–III, VII and XIII are cytosolic; IV and XV are glycosylphosphatidylinositol-anchored proteins; VA and VB are mitochondrial; VI is secreted into saliva and colostrum; IX, XII and XIV are transmembrane proteins; and VIII, X and XI have no catalytic activity) [205,208]. Specifically, only carbonic anhydrases IV, V, VII and XIV have been identified throughout the human and murine central nervous system (CNS) [209,210,211].

As previously mentioned, carbonic anhydrases play a key role in the control of pH by maintaining the intra- and extracellular balance between CO_2_, HCO_3_^−^ and H^+^ at the level of the whole organism (for example, respiratory and renal functions), in the BBB, in neurons and glia and in the interstitial fluid in the brain [212]. In the brain, the activation of the GABAA receptor opens channels that are more permeable to Cl^−^ than to HCO_3_^−^, leading to a membrane hyperpolarization and GABA-induced inhibition. However, as seen in epilepsy, a prolonged activation of GABAA receptors can provoke an excessive accumulation of Cl^−^ ions in postsynaptic neurons; this depolarizes the postsynaptic neurons since the intracellular HCO_3_^−^ is constantly replenished due to the intracellular carbonic anhydrases activity. In conclusion, GABAergic transmission can exert both seizure-suppressing and seizure-promoting actions, respectively [213]. Other studies have also reported that brain carbonic anhydrases play crucial roles in long-term synaptic transmission and memory storage via a similar pathway [207,214].

### Carbonic Anhydrase, Seizures and Epilepsy

Abnormal levels or activity of the membrane-associated carbonic anhydrases have been related with different diseases such as obesity (VA and VB), retinitis pigmentosa, glaucoma (IV and XII), cancer (IX and XII) and epilepsy (XIV) [215,216,217,218,219]. Considering that epilepsy is a neurological disorder related to an imbalance between inhibitory and excitatory neurotransmission [220,221], there is a high probability that epilepsy could also be associated with fast alterations in extracellular ionic composition [212,222].

The relation between carbonic anhydrases and seizures has been supported since the 1950s by several clinical and experimental studies reporting that carbonic anhydrase inhibitors such as acetazolamide [223,224], methazolamide [225], topiramate [226,227] and zonisamide [228,229] have anticonvulsant effects. This effect has been attributed to CO_2_ retention, secondary to inhibition of the red cell and brain enzymes. Moreover, other mechanisms have been suggested, involving: (a) a blockade of sodium channels and kainate/AMPA receptors; and (b) an enhancement of the GABAergic transmission [230]. However, the potential of the previously mentioned inhibitors in the treatment of epilepsy and other neurological disorders is not fully understood since the precise carbonic anhydrase isoforms involved remain unknown [231,232]. During the last decade, the carbonic anhydrase-VII isoform has become a validated target given its brain distribution (mainly expressed in regions as cortex, hippocampus and thalamus) [211,233,234] and its previously mentioned involvement in neuronal excitation by enhancing bicarbonate-driven GABAergic excitation during intense GABAA receptor activation. In this sense, Ruusuvuori et al. [235] conducted a study on carbonic anhydrase-VII knockout mice where they addressed the role of carbonic anhydrase-VII in HCO_3_^−^-dependent depolarizing GABA responses and in the generation of experimental febrile seizures induced by hyperthermia. To date, this animal model has provided new opportunities to examine how this neuron-specific isoform modulates excitability.

Currently, the main scope is to design, synthesize and evaluate new carbonic anhydrase-VII inhibitors [236,237]. In this sense, De Luca et al. [238] performed a virtual screening to find such compounds. To do so, they built pharmacophore models from crystal structures of two well-known carbonic anhydrase inhibitors in complex with human carbonic anhydrase-VII. A merged pharmacophore model was obtained and, subsequently, a library of thousands of compounds was screened; new chemical entities displaying significant carbonic anhydrase inhibitory effects in the nanomolar range were identified. More studies are needed to complete the anticonvulsant profile of the new compounds in the treatment of febrile seizures [235]. However, the role that anhydrase-VII might have in drug-resistant epilepsy, if any, is still unknown.

## 6. Erythropoietin

The hormone erythropoietin is an ~30 kDa multifunctional glycoprotein that has a crucial role in erythropoiesis by preventing apoptosis and inflammation (caused by certain circumstances such as hypoxia, toxicity or injury), and by stimulating the proliferation of erythroid progenitors and the production of red blood cells in the bone marrow [239,240]. It has been detected in several tissues such as liver (during fetal development), kidney [241,242], heart [243], uterus [241,244] and brain [241,245]. In rodent and human CNS, erythropoietin is expressed in astrocytes and neurons while its receptor (EPOR; a transmembrane receptor member of the type I cytokine superfamily which is pre-formed as homodimers on the cell surface) is expressed in endothelial cells, microglia, astrocytes, oligodendrocytes and neurons [246]. The vast extension of hormone and receptor activity is related to endogenous potent neuroprotective effects [247,248,249,250,251,252].

The erythropoietin-mediated neuroprotective effects have been reported in diverse studies of ischemia, parkinsonism, brain trauma, seizures and epilepsy [253,254,255,256,257,258,259,260,261,262], as well as its therapeutic effects, in different neurological disorders, such as stroke [263], schizophrenia [264] and Parkinson’s disease [265], among others.

The neuroprotection induced by erythropoietin has been associated with several mechanisms, including: (1) neurogenesis [266]; (2) protection against glutamate-induced neuronal damage [267]; (3) antioxidant and anti-inflammatory activity by inhibiting the nuclear factor-KB (NFKB) [268]; (4) antiapoptotic activity [269]; (5) BBB protection [270]; and (6) microglia modulation [271]. In contrast, other studies have reported that erythropoietin augments neuronal activity via Ca^2+^ channels activation [272] and increases free cytosolic concentrations of Ca^2+^ [273]. This suggests that the erythropoietin-induced neuroprotective effect can only be produced under certain cerebral conditions.

### Erythropoietin and Epilepsy

In acute seizures models (PTZ and KA), of induced SE (pilocarpine or electrical) and of TLE, the modulatory effect of erythropoietin has been suggested as a strategy able to: (1) reduce the seizure severity [274]; (2) protect against the BBB disruption and the subsequent microglial activation [260,275]; (3) prevent aberrant neurogenesis and granule cell dispersion [260,274]; (4) inhibit proinflammatory mediators [276]; (5) decrease neuronal death and apoptosis [260,277,278]; and (6) provide protective effects against epileptogenesis [260].

Based on the previously mentioned neuroprotective and anti-inflammatory effects of erythropoietin, some studies have focused on the antiepileptogenic ability of erythropoietin-derived mimetic peptides. In this direction, Seeger et al. [279] administered the peptide pyroglutamate helix B surface peptide (pHBSP) after the electrical induction of a self-sustained SE, observing enhanced hippocampal cell proliferation, neuronal differentiation and cell survival. However, the number of rats presenting spontaneous recurrent seizures was not modified. Simultaneously, Zellinger et al. [280] reported the effects of epotris, another erythropoietin-derived peptide devoid of erythropoietic activity. Here, they evaluated the histopathological consequences of electrically-induced SE, reporting limited in vivo effects on cell consequences caused by prolonged seizures. Future studies, using the appropriate animal model and the optimum treatment parameters, are necessary to support the erythropoietin-derived peptide mimetic design as a reliable therapeutic strategy to prevent the onset and progression of epilepsy.

Regarding to erythropoietin receptor, Sanchez et al. [252] suggested that optimal neuroprotection by erythropoietin requires an elevated expression of its receptor in neurons. This idea was later supported by Ott et al. [281], reporting the upregulation of erythropoietin receptors in specific hippocampal areas of a drug-resistant epileptic patient submitted to surgery. Some of the proposed mechanisms underlying this effect can be associated with erythropoietin’s ability to inhibit caspase-3 [282] and to activate JAK-2, ERK-1/-2 and Akt pathways; this activation is associated with elevated Bcl-XL levels in neurons [283]. In consequence, the increased amount of Bcl-XL can downregulate the expression of pro-apoptotic proteins such as Bax and Bid [284]. These results confirm that erythropoietin protects neurons and suggests that this can result in further suppression of neuronal apoptosis by regulating the balance between pro- and anti-apoptotic Bcl-2 family proteins.

The notion that seizure activity represents a condition that induces brain hypoxia (a discrepancy between an adequate supply of oxygen through blood vessels cells and consumption) and ischemia is well established [285,286]. Experimental evidence demonstrates that both processes enhance glutamate release [221,287,288] and facilitate neuronal cell death [289,290], membrane depolarization [291,292], subsequent epileptic seizures and overexpression of the *MDR-1* gene that encodes the multidrug efflux transporter P-glycoprotein (condition associated with drug-resistance) [293,294]. In addition, this augmented expression of P-glycoprotein can also facilitate cell membrane depolarization as a consequence of increased amounts of intracellular free radicals and Ca^2+^ [295,296].

On the other hand, seizure activity is also known to activate the hypoxia-inducible Factor 1 (HIF-1), which modulates the expression of hypoxically regulated genes such as those encoding erythropoietin and the vascular endothelial growth factor (VEGF) [297,298,299,300,301]. These results suggest that, in addition to the erythropoietin neuroprotective properties, the overexpression of HIF-1α and VEGF in neurons can also be a mechanism of cerebral protection in epilepsy. Given the previous findings regarding the neuroprotective, anti-inflammatory and antiepileptogenic effects of erythropoietin, it is reasonable to suggest that the development of erythropoietin-derived mimetic peptides could be a therapeutic target to treat epilepsy and drug-resistant epilepsy.

As the human recombinant erythropoietin was approved by the FDA as a safe and highly effective compound in the treatment of anemia in adults and children with renal failure, cancer and prematurity [302], further studies are necessary to establish the protective effect of recombinant erythropoietin in the brain once the epileptogenesis process has started, without causing undesirable side effects.

## 7. Copines

Copines integrate a small family of evolutionary conserved proteins able to bind to phospholipids in a calcium (Ca^2+^)-dependent manner [303]. They are found in diverse eukaryotic organisms and are characterized by two C2 domains in the N-terminal region of the protein followed by an “A domain” in the C-terminal region of the same protein. Following the “A domain”, copines have a variable-length C-terminal domain, which may confer unique characteristics to the different copine family members [304,305,306]. To date, there are nine copines (1–9) identified in mammals, ubiquitously expressed in diverse tissues such as heart, lungs, spleen, kidneys, prostate and brain [305,307,308,309,310,311,312,313]. Although the precise function of copines in mammalian cells remains unclear, evidence suggests that they are involved in regulating signaling pathways [306], in membrane trafficking [314] and in morphogenetic processes associated with the maturation of retinal synaptic architecture [315].

### Copine 6 and Epilepsy

Copine 6 (also known as N-copine (neuronal-copine)) expression is almost restricted to the brain and it was first identified by Nakayama et al. in 1998 [307]. In that study, they reported that, in the hippocampal neurons, copine 6 mRNA expression is upregulated by experimental induction of seizures (via kainate injection) in an NMDA-dependent manner or after long-term potentiation induction. Later studies not only confirmed that copine 6 is expressed in the brain, but it is also involved in the translation of initial calcium signals into dendritic spine morphology changes to promote synaptic plasticity [316,317]. However, the precise functional role of copine 6 in the brain is not yet fully understood.

Diverse studies have described that certain patterns of dendritic spine disruption (e.g., structure, function, distribution and connectivity) may lead to altered neuronal circuits and become an underlying cause of many psychiatric and neurologic diseases such as Fragile X and Down syndrome [318,319], autism [320], Alzheimer’s disease [321,322], Parkinson’s disease [323] and epilepsy [324,325], among others.

In the specific case of TLE, one of the main forms of drug-resistant epilepsy, clinical and experimental studies point out to the altered balance between inhibitory and excitatory circuits [220,286,326,327] as a factor that facilitates aberrant plastic changes among hippocampal cells due to the constant uncontrolled occurrence of seizures and the subsequent long-term potentiation of synaptic transmission [328,329]. Therefore, we can conclude that plasticity is not necessarily a positive process in epilepsy and elucidate a possible relationship between epilepsy and copine 6 based on the notion that this protein is involved in synaptic plasticity due to its association with long-term potentiation [307,330].

Almost two decades later, Zhu et al. [331] published the first study focused on the expression of copine 6 in the brain tissue of epileptic patients. Here, they performed quantitative real-time PCR, Western blot and immunofluorescence analysis to evaluate the expression and distribution of this copine in the brain tissue of drug-resistant epileptic patients and pilocarpine-induced epileptic rats. Their results are the first to show the following findings: (1) copine 6 expression is significantly increased in the epileptic brain of the drug-resistant patients; (2) copine 6 mRNA and protein are significantly increased once the animals develop pilocarpine-induced spontaneous recurrent seizures; and (3) copine 6 protein is mainly located in neurons. These data suggest a new therapeutic target for the treatment of drug-resistant epilepsy. Moreover, although increased expression of copine 6 is observed after epilepsy development, further studies are necessary to determine if copine 6 is also involved in the epileptogenesis process.

In addition, it was reported there exists an increased interaction of adenylate kinase 5 (AK5) with copine 6, in TLE patients and experimental epileptic rats, where the AK5-copine 6 complex could play an important role in regulating epileptic seizures and epileptogenesis [332], suggesting a new therapeutic targets for the treatment of epilepsy, particularly drug-resistant epilepsy.

## 8. The Complement System

The complement system is a group of sequentially reacting proteins that, upon activation, mediate a number of biological reactions important in host defense [333]. Nowadays, it is well accepted that complement is a critical part of the innate immune response, playing a key role in host homeostasis, inflammation and defense against pathogens [334].

The complement system is comprised of numerous soluble proteins and membrane expressed receptors and regulators that operate in plasma, in tissues, on cell surface and even within the cell [335,336]. It is composed of more than 40 proteins which activate a cascade of molecular events leading to multiple effector mechanisms to protect the host in response to a wide variety of stimuli (order of activation: C1, C4, C2, C3, C5, C6, C7, C8 and C9) [336].

### 8.1. Complement System Activation

**Figure 3:**
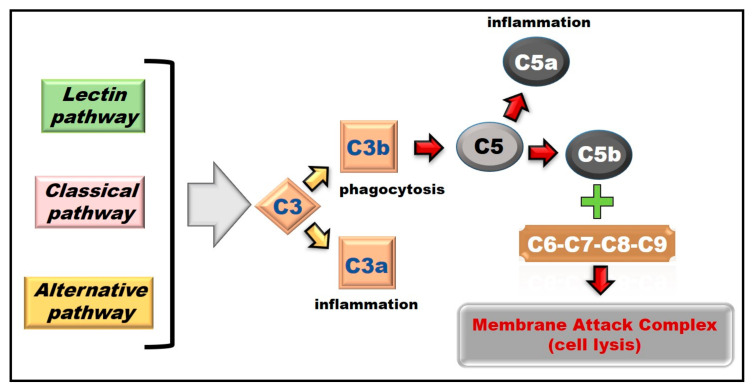
Overview of the complement system. Complement activation can be initiated by three distinct pathways: the classical, lectin and alternative pathways. Although each one is activated in response to different molecules, all of them manage to activate complement factors (C3 and C5) and promote the release of anaphylatoxins (C3a and C5a) associated with inflammatory processes. Complement activation ends with the membrane attack complex (MAC; C5b–C9) formation which can then cause targeted cell lysis.

The complement system can be initiated depending on the context by three distinct pathways: (a) classical; (b) lectin; and (c) alternative. Each of them leads to a common terminal pathway, a non-enzymatic pathway, in which the terminal components are recruited sequentially from the fluid phase to form a large pore, that disrupts the membrane to cause target lysis (Figure 3) [336,337].

The classical pathway activation is determined by the binding between antibody complexes and the complement factor C1 [333,337]; the lectin pathway activation is due to the interaction with certain class of carbohydrate residues from pathogenic microorganisms [333,337]; and the alternative pathway activation is determined by the presence of foreign surfaces uncommon to the host cells [333,337].

All these three pathways overlap in the cleavage of C3 into its active fragments (C3a and C3b), to later continue with the activation of the complement cascade and to lead to the formation of the active fragments of C5 (C5a and C5b) [333,338] and the final assembly of the Membrane Attack Complex (MAC), a transmembrane pore consisting of C6–C7–C8–C9 complement factors, which will allow targeted cell lysis (Figure 3) [333,339,340].

C3a and C5a are molecules known as anaphylatoxins associated with inflammatory response [338,341,342]. They have the ability to function as chemoattractants to recruit immune cells to the complement activation site promoting the release of inflammatory mediators [338,342,343]. The C5a anaphylatoxin exerts its effects through binding to the C5a complement receptor 1 (C5ar1), which belongs to the G protein-coupled receptors (GPCR) family [338,343,344].

### 8.2. Complement System and Epilepsy

Complement causes pathology when regulation fails, leading to excessive activation that drives inflammation and tissue damage, which may contribute to seizure generation and epilepsy development.

Gene expression studies have demonstrated overexpression of various complement proteins in surgically removed tissue from patients with TLE [345,346,347,348] as well as in rodent TLE models [348,349]. Specific genetic polymorphisms in the C3 promoter region of mesial TLE patients suggested the involvement of the complement system in the genetic susceptibility to febrile seizures and to epilepsy [347]. In addition, increased immunoreactivity of multiple complement factors, such as C1q, C3, C4 and MAC, have been documented on activated microglia and select neurons in brain tissue from TLE patients and rodent TLE models [348,350], where the presence of MAC strongly suggests complement cascade activation in epileptic tissue. Another important finding is that sequential intrahippocampal injection of individual proteins of MAC (C5b6, C7, C8 and C9) in awake-freely moving rats, induce both behavioral and electrographic seizures as well as neurodegeneration [351].

Animal studies using transgenic mice have shown that the complement system contributes to seizures. Thus, *C3-deficient mice* develop significantly fewer behavioral seizures following Theiler’s virus infection than *wild-type mice* [352], while, in *C6-deficient rats*, the kindling development was delayed [353].

In *C5ar1-deficient mice*, it was demonstrated that complement receptor C5ar1 contributes to the acute inflammatory response elicited by infiltrating immune cells following pilocarpine-induced SE [354]. These findings suggest multiple mechanisms for the protective effects of C5ar1 pharmacologic inhibition or genetic ablation [354,355].

In this sense, targeting the complement could be a novel viable therapeutic strategy in epilepsy. Specifically, the therapeutic potential of the inhibition of the C5ar1 has been studied. The inhibition of C5ar1 by PMX53 antagonist was found to have anticonvulsant effects in the 6 Hz and corneal kindling model (acute seizure models), as well as in the intrahippocampal kainate model (chronic seizure model) [355]. Furthermore, inhibition of C5ar1 after pilocarpine-induced SE, or its genetic deletion, lessened seizure magnitude, protected hippocampal neurons from degeneration and reduced SE associated mortality [355]. It has been suggested that inflammatory cytokine expression and modulation of microglial potassium channels are affected by C5ar1 absence or inhibition by PMX53, with possible association to neuroprotective effects [355]. PMX53 efficacy in these models set the possibility of being effective for drug-resistant seizures types. The limitation of PMX53 is its short half-life [355]; therefore, new brain-permeable C5ar1 inhibitors with an improved pharmacokinetic profile need to be developed.

The complement receptor C5ar1 expression on microglia has been reported upregulated following kainate-induced seizures, which is controlled by the *Fosb gene*, a mediator of transcriptional factor, activator protein-1 (AP-1) [356]. Therefore, under excitotoxic conditions, *Fosb gene* products contribute to microglial activation in the brain through regulation of microglial C5ar1 expression [356].

Additionally, classical complement pathway markers (C1q and iC3b) were altered alongside the phagocytic signaling molecules (Trem2 and Pros1) in human brain samples obtained from patients with focal cortical dysplasia [357], suggesting that aberrant phagocytic signaling and complement activation occurs in human drug-resistant epilepsy [357]. The alteration of phagocytic pathways may contribute to unwanted elimination of cells/synapses and/or impaired clearance of dead cells, whereas the classical complement pathway may be a candidate mechanism contributing to epileptogenic remodeling of the synaptic circuitry associated with human drug-resistant epilepsy [357]. In epileptic animals, SE induced a long-lasting increase in C1q, C3 and iC3b proteins in the hippocampus that correlated with higher seizure frequency [358]. However, how the hyperactivation of the classical complement pathway directly contributes to the molecular/neuronal and/or clinical pathology of SE and TLE remains unknown.

Complement factors may also serve as biomarkers for the epilepsy diagnosis since increased serum levels of these proteins have been found in epileptic patients [359]. The serum level of C3 complement protein was found to be higher in untreated epileptic patients than in healthy controls or treated epileptic patients, suggesting complement involvement in seizures generation [359]. Recently, changes were identified in the plasma concentration of a panel of complement analytes (including markers of classical, alternative and terminal activation pathways) between patients with epilepsy and controls, controlled and uncontrolled epilepsy and with certain AEDs [360].

The first successful therapeutic treatment to inhibit the complement system consists of targeting the terminal pathway with the use of an anti-C5 antibody, named eculizumab [361]. Eculizumab (Soliris™, Alexion Pharmaceuticals) has proven safe and effective in managing paroxysmal nocturnal hemoglobinuria (PNH) and atypical hemolytic uremic syndrome (aHUS) [361,362,363]. Eculizumab treatment blocks the cleavage of C5 and prevents the formation of the MAC but leaves the rest of the complement system intact [361]. Although eculizumab and many other complement inhibitors have not been tested in epilepsy, there are questions about the safety of chronic inhibition of complement components and which key proteins to block in all complement pathways, since the complement system is an important part of the immune system.

These data add further evidence to the role of complement dysregulation that could mediate relevant changes related to seizure generation and to the pathogenesis of epilepsy or drug-resistant epilepsy.

## 9. Transient Receptor Potential Vanilloid Type 1 (TRPV1)

Transient receptor potential vanilloid type 1 (TRPV1) is a ligand-gated nonselective cation channel and member of the vanilloid TRP family. In the chromosome 17p13 is located the Trpv1 gene, which encodes a 95-kDa protein containing 839 amino acid residues [364]. TRPV1 has six transmembrane domains (TM), presenting a pore region located between TM5 and TM6 domains. The N-terminal tail allows binding of calmodulin and ATP to modulate TRPV1 activation while C-terminus contains a TRP domain and binding sites for phosphatidylinositol 4,5-bisphosphate (PIP2) and calmodulin [364].

TRPV1 is a nonselective cation channel with permeability to calcium (Ca^2+^), protons (low pH) [365], polyvalent cations, anandamide and arachidonic acid metabolites [366,367].

When these channels are stimulated, they increase permeability to Na^+^ and Ca^2+^, therefore increasing neuronal excitability [368,369]. Furthermore, they respond to stimuli such as noxious heat [368], oxidative stress and vanilloids (i.e., capsaicin, olvanil and resiniferatoxin) [368]. In addition, the mitochondrial activation of TRPV1 induces an increase of Ca^2+^ concentration, mitochondrial reactive oxygen species production and MAPK activation, which correlates with an augmented migration of microglia [370].

TRPV1 was first reported in sensory neurons of the dorsal root ganglion, nodose ganglia and trigeminal ganglia [364]. In the brain, it is found in regions of the hypothalamus, cerebellum, cerebral cortex, striatum, midbrain, hippocampus and substantia nigra [371]. The human hippocampus and cortex express relatively high levels of TRPV1 mRNA [371]. On the other side, the subcellular localization of TRPV1 is in the inhibitory and excitatory synapses of the dentate molecular layer (ML), in the postsynaptic dendritic membranes in the mouse hippocampus [372,373]. Moreover, they are expressed in nonneuronal cells such as epidermal keratinocytes [374], urinary bladder epithelial cells [375], pancreatic islet beta cells [376], neutrophil granulocytes [377], human brain endothelium [378] and human peripheral blood [379].

Current knowledge demonstrates that activation of TRPV1 mediates synaptic plasticity, facilitating long-term potentiation and suppressing long-term depression [380], neurotransmitter release [381,382,383] and the enhancement of synaptic excitation [384]. TRPV1 activation regulates spontaneous glutamate release in an activity-dependent manner in rodent brain [382,385] and human cortical tissue [369].

Additionally, in recent years, extensive research has been directed towards studying TRPV1, because it is also involved in the pathophysiology of diseases such as schizophrenia [386], stroke [387], pain [388], depression [389], anxiety [390], Parkinson’s disease [391] and epilepsy [392].

### TRPV1 and Epilepsy

Because TRPV1 regulates neuronal excitability, this channel could be a promising therapeutic target for the treatment of epilepsy. For this reason, we provide a brief description of the TRPV1 effects in preclinical and clinical models of epilepsy.

In *TRPV1 knockout mice*, there was an observed reduction in the susceptibility to hyperthermic seizures, as well as a delay in seizure latency, short seizure duration and a decrease in seizure severity compared to *wild type mice* [393], suggesting that the reduction of TRPV1 expression paralleled a decreased susceptibility to febrile seizures. Furthermore, *TRPV1 knockout mice* showed a decrease in susceptibility to generalized clonic-seizures induced by PTZ [394].

In vitro studies showed that capsazepine (CPZ, a TRPV1 antagonist) suppressed the epileptiform activity induced by 4-aminopyridine (4-AP) in mice hippocampal slices. In contrast, capsaicin (a TRPV1 agonist) promoted epileptiform activity [395]. These results support a role for TRPV1 in the suppression of epileptiform activity, indicating that antagonism of TRPV1 could be a target for effective acute suppression of seizures [395].

Other studies have also reported the antiepileptic actions of the TRPV1 antagonists. Regarding *genetically epilepsy-prone rats* (*GEPR-3s*), CPZ (a TRPV1 antagonist) reduced seizure severity in a dose-dependent manner in male rats and in female rats CPZ suppressed seizure susceptibility [396]. With the application of CPZ and other TRPV1 antagonists such as 5-iodoresiniferatoxin and α-Spinasterol, seizures were suppressed, regardless of the method of induction (4-AP, PTZ, maximal electroshock test, 6 Hz stimulation) [392,395,397,398,399,400]. CPZ also decreased intracellular reactive oxygen species production, mitochondrial membrane depolarization, apoptosis and the levels of caspases 3 and 9, in the hippocampus and dorsal root ganglion of rats in the 4-AP model [392,400] where inhibition of TRPV1 activity can result in neuroprotective effects in the epileptic neurons.

On the other side, it has been reported an increased expression of protein and mRNA levels of TRPV1 in the hippocampus, mainly in CA1 and CA3 areas, during acute and chronic phases of pilocarpine-induced SE [401,402].

Regarding TRPV1 agonists, capsaicin enhanced spontaneous excitatory postsynaptic currents (EPSC) frequency and TRPV1 protein expression in the dentate gyrus of mice with TLE [384]. N-oleoyldopamine (a TRPV1 agonist), increased the incidence and duration of generalized clonic-tonic seizures as well as decreased the latency of myoclonic seizures, both in the PTZ- and amygdala-kindling models [403]. However, AMG-9810 (a TRPV1 antagonist), reduced not only the duration of generalized clonic-tonic seizures in the PTZ-kindling model but also the duration of afterdischarges in the amygdala-kindling model [403].

Patients with mesial TLE showed upregulation of mRNA and protein expression levels of TRPV1 in the temporal cortex and hippocampus [404]. TRPV1 were mainly distributed in the cell bodies and dendrites of neurons and in astrocytes and co-localized with glutamatergic and GABAergic neurons [404], suggesting that the overexpression and distribution patterns of TRPV1 might be involved in the pathogenesis and epileptogenesis of human TLE [404].

Taken together, these findings support the role of TRPV1 in the etiology of epilepsy. Systemic administration of TRPV1 antagonists seems to be a novel therapeutic target for epilepsy, given their antiepileptic, antiepileptogenic or neuroprotective effects in preclinical models.

The effectiveness of TRPV1 antagonists should be studied with caution, as there are no chronic studies that show pharmacokinetic profile, efficacy and tolerability in preclinical models of epilepsy. Furthermore, there are currently no clinical trials with TRPV1 antagonists demonstrating its effectiveness in epileptic patients.

In the future, it will be interesting to investigate the TRPV1 involvement in drug-resistant epilepsy, in both preclinical models and surgically resected human tissue, where these studies may lead to knowing whether TRPV1 participates in the pathophysiology of drug-resistant epilepsy and if they could be a new therapeutic target.

## 10. Galanin and Galanin Receptors

Galanin is a 30 amino acid residue long endogenous neuropeptide that presents an N-terminal core fragment formed by residues of Gly1, Trp2, Asn5, Tyr9 and Gly12 and is associated with maintaining high affinity with galanin-receptors [405].

Galanin is widely distributed in both the peripheral and CNS of several mammalian species [406]. In the CNS, it is an inducible neuropeptide which concentration can be increased (2–10-fold) by physiological regulators such as estrogen [407], vasoactive intestinal peptide [408], thyroid hormone [409], progesterone [410], nerve growth factor [411] and brain-derived nerve growth factor [412].

Galanin can be co-expressed with neurotransmitters such as acetylcholine, serotonin, glutamate, GABA, noradrenalin and dopamine [413], as well as with neuropeptides such as encephalin, neuropeptide Y, substance P, vasopressin, calcitonin gene-regulated peptide and gonadotropin-releasing hormone [414].

Galanin participates in biological processes such as feeding [415], nociception [416], nerve regeneration [417], arousal/sleep regulation [418], sexual behavior [419], learning [420], memory [421], neuroendocrine release [422] and gut secretion and contractility [423].

In addition, it is involved in pathological processes such as ischemic brain damage [424], chronic stress [425], nerve injury [426], multiple sclerosis [427] and Alzheimer’s disease [428].

Galanin actions are mediated through three G-protein-coupled receptors, namely GalR1, GalR2 and GalR3, which are linked to different signaling cascades [429]. These galanin-receptors have extensive distribution throughout the CNS, mainly in the hippocampus, hypothalamus, locus coeruleus neurons and medulla oblongata [430,431].

The GalR1 contains 349 amino acids and it is located on chromosome 18q23 [432]. The activation of GalR1 induces the hyperpolarization of the presynaptic terminals by opening ATP-dependent K^+^ channels, thus inhibiting glutamate release in the hippocampus [433,434]. Additionally, GalR1 has been shown to directly close voltage-gated Ca^2+^ channels which could also hyperpolarize the presynaptic membrane and prevent glutamate release [435]. GalR1 has also been involved in the regulation of the transcription factors cAMP response element-binding protein (CREB) and the immediate early gene c-fos, in specific brain regions [436,437].

The GalR1 and GalR3 receptors act via a G_αi/o_-protein, resulting in the activation of G protein-regulated inwardly rectifying K^+^ channels, as well as in a decrease of adenylate cyclase (AC) activity and cytosolic cAMP levels [438,439,440].

The GalR2 is conformed by 387 amino acids and located on the chromosome 17q25.3 [441]. GalR2 signals through several classes of G-proteins, the most common being the stimulation of the phospholipase C via G_αq/11_-protein family, which increases inositol phosphate hydrolysis, mediating the release of Ca^2+^ into the cytoplasm from intracellular stores and opening Ca^2+^-dependent chloride channels [441]. GalR2 activation via G_αi/o_-protein induces the regulation of CREB in mouse dentate gyrus [436] as well as the stimulation of MAPK/ERK1/2 pathway in different cell types [439,442,443,444].

GalR3 contains 368 amino acids and it is located on chromosome 22q12.2–13.1 [445]. The role of GalR3 in the brain is less studied. However, the evidence suggest that GalR3 activation increases neural stem cells viability [446]. GalR3 activation also affects the phosphorylation of CREB [447] and has a more restricted expression to hypothalamus and areas of the mid- and hindbrain, as compared with the other galanin-receptors [448].

In general, the galaninergic innervation is abundant in the hippocampus, and it has been suggested that this peptide modulates the activity of principal neuronal populations in the dentate gyrus and CA3-CA1 through the activity of GalR1 and GalR2 [449].

### Galanin and Galanin-Receptors in Epilepsy

Galanin is a modulator of classical neurotransmitters and is present in brain areas intimately involved in epilepsy, which makes galanin an interesting therapeutic target to study in relation to its involvement in the generation and spread of seizures, with the end goal of developing novel approaches for the treatment of epilepsy.

In this context, a depletion of galanin immunoreactivity has been reported in nerve fibers of all hippocampal areas after SE, induced by stimulation of the perforant path-pathway [450]. However, in the SE induced by KA, it was demonstrated that galanin was significantly upregulated after SE. These changes were transient, most often limited to 6–24-h post-seizure and returning to baseline by 72 h [451]. On the other hand, galanin induced a dose-dependent antiepileptic effect in the picrotoxin-kindling model when administered into the lateral brain ventricle, hippocampus, caudate nuclei, substantia nigra reticulata and nucleus accumbens [452].

In vitro studies with human hippocampal slices obtained from patients with TLE, showed that galanin applied directly to slices had no significant effect in stimulation-induced EPSPs (excitatory postsynaptic potentials) in dentate gyrus and CA1. Autoradiographic binding revealed the presence of galanin-receptors and the functional receptor binding assay showed impaired functionality of these receptors, suggesting that the signaling pathways could be altered [453]. In addition, in TLE patients, a pathogenic missense mutation in the galanin gene has been identified [454].

All previous results suggest that alterations in galanin-receptor binding and downstream signaling could have a role in the generation of seizures [454].

It has been found that GalR1 mediates galanin protection from seizures and seizure-induced hippocampal injury in models of limbic SE. GalR1 knockout mice present severe seizures, profound injury in the CA1 pyramidal cell layer, as well as injury to hilar interneurons and dentate granule cells, in Li-pilocarpine and electrical perforant path stimulation (PPS) models [455]. These mice also presented spontaneous seizures ranging from facial movements to SE [456], which is probably associated with changes in the distribution and expression levels of several neuropeptides in some principal hippocampal neurons, such as a strong upregulation of galanin expression only in neurons in the polymorph layer of the dentate gyrus [457]. However, *GalR2 knockout mice* showed no change in seizure latency or the number of animals with seizures versus *wild type mice*, in PTZ and flurothyl models [458]. In the electrical PPS model, *GalR2* *knockout mice* showed neuronal damage in the dentate hilus, hilar injury and increase of seizure severity [459].

*GalR1-/-mice* (mice with an insertional inactivating mutation within the gene encoding GalR1) showed more susceptibility to tonic-clonic seizures in response to bright overhead lighting or handling [456], and developed spontaneous seizures of partial onset with secondary generalization [460]. The above indicates a critical role for GalR1 in the regulation of the antiseizure activity of galanin.

Different GalRs agonists and positive allosteric modulators have shown anticonvulsive and antiepileptogenic effects in different animal models. For example, M617 (a GalR1 agonist) administered during 24 h by intrahippocampal infusion in wistar rats, increased the after-discharge threshold and delayed the onset of seizures, but did not block the occurrence of generalized seizures, in the rapid kindling model [461]. These results suggest that the activation of GalR1 decreased the excitability of the hippocampus and delayed, although it did not prevent, the epileptogenesis induced by the kindling. On the other hand, galanin(1-29) (a GalR1/2 agonist) and galanin(2-11) (a GalR2 agonist) completely prevented the occurrence of full kindled seizures [461], where both agonists could have an antiepileptogenic effect possibly mediated by GalR2. However, galmic (a nonpeptide galanin-receptor agonist) had a dose dependent effect, decreasing seizures and reducing the severity and duration of spikes of SE induced for the perforant path stimulation in the hippocampus of mice [462].

The galanin analogs (Gal-(K) 4, Gal-B5, Gal-B6, and Gal-B7) showed some protection against seizures in the 6 Hz corneal stimulation model. However, only Gal-B2 (NAX-5055) provides 100% protection against seizures in the same model [463], and in the Frings audiogenic seizure susceptible mouse model [464]. Furthermore, NAX-5055 reduced the vesicular release of glutamate and increased the extracellular level of GABA in cerebellar, neocortical and hippocampal slices of rats [465], which partly explains the anticonvulsant effect of NAX-5055 observed in epilepsy models [465].

The GalR1 agonist, galnon, seems to reduce the severity and increased the latency of PTZ-induced seizures [466]. While its intrahippocampal injection shortened the duration of self-sustaining SE induced by perforant path stimulation [466]. NAX 810-2, a GalR2 agonist, blocked in a dose-dependent manner seizures induced by corneal kindling and 6 Hz stimulation [467]. Although CYM2503 (a GalR2-positive allosteric modulator) did not seem to block the establishment of SE, it increased the latency to seizure and to SE, and, more importantly, it appeared to decrease mortality dramatically, in the Li-pilocarpine model [468]. CYM2503 also attenuated electroshock-induced seizures [468].

Another line of evidence that suggests an antiseizure effect due to galanin is the overexpression of galanin in animal models of epilepsy.

Overexpression of galanin in dentate granule cells and hippocampal and cortical pyramidal neurons delays seizure generalization during hippocampal kindling [469]. Galanin overexpressed ectopically (GalOE) in the olfactory bulb of mouse modulated high frequency excitatory synaptic transmission, increased latency to behavioral convulsions and increased the number of stimulations necessary to reach the fully kindled state [470]. This finding suggests that galanin overexpressed outside of the hippocampal formation has a significant effect, which could contribute to inhibiting seizures in the brain [470]. Furthermore, overexpression of the galanin through viral vectors suppressed generalized seizures in kindled rats [471]. Adeno-associated virus (AAV)-mediated expression and constitutive secretion of galanin, prevented neuronal cell death and blocked limbic seizure activity induced by KA and kindling [472,473,474], demonstrating an antiseizure effectiveness of galanin.

The evidence shown suggests that galanin and galanin-receptors represent promising novel targets for drug development for the treatment of epilepsy. However, galanin and galanin-receptors agonists have low bioavailability due to rapid degradation by peptidases, low permeability through the BBB, and time to peak effect of 30 min; they are biologically unstable, with a half-life less than 1.2 h; and there are currently no agonists and antagonists with a high level of specificity. Only NAX 5055 (with high affinity for GalR1-GalR2) was found to be biologically active after intravenous, intraperitoneal and subcutaneous administration, and its efficacy was associated with a linear pharmacokinetic profile [464]. For this reason, the employment of AAV vectors for expression and constitutive secretion of galanin is an important tool for the development of antiepileptic gene therapy. The study of galanin and galanin-receptors in epilepsy should be focused on promoting the search for new therapeutic targets as well as the development of new drugs such as peptidomimetics of galanin, non-peptide agonists or positive allosteric modulators. Finally, it would be interesting to investigate their potential use as therapeutic targets in drug-resistant epilepsy.

## 11. Melatonin and Melatonin-Receptors

Melatonin (N-acetyl-5-methoxytryptamine, an indoleamine derivative of serotonin) is a neurohormone synthesized and secreted during the night by the pineal gland [475]. Tryptophan first converts to serotonin, and then melatonin synthesis takes place via a two-step pathway: the first step is catalyzed by arylalkylamine *N*-acetyltransferase enzyme to *N*-acetylserotonin and the cycle ends with the synthesis of melatonin by enzyme hydroxyindole-*O*-methyltransferase [476].

The melatonin secretion pattern is regulated by the suprachiasmatic nucleus of the hypothalamus [477]. Melatonin is also produced by other organs such as the retina [478], skin [479], gastrointestinal tract [480], bone marrow [481] and lymphocytes [482].

Melatonin regulates several mammalian biological processes such as circadian rhythm [483], sleep [484], reproduction [485], retinal physiology [478], glucose homeostasis [486], memory [487], blood pressure control [488], immune function [489] and free-radical scavenging [490]. Furthermore, other results suggest that melatonin has antinociceptive [491], antidepressant [492], antioxidant [493] and anxiolytic effects [494].

Melatonin acts on a receptor family composed of MT_1_, MT_2_ and MT_3_. The melatonin-receptors have been localized in areas of the nervous system and peripheral tissues of both rodents and humans [495]. Their distribution in the brain includes the cerebellum, occipital cortex, parietal cortex, temporal cortex, thalamus, frontal cortex, striatum, substantia nigra, ventral tegmental area, nucleus accumbens, caudate-putamen, hippocampus and suprachiasmatic nucleus [495].

The MT_1_ receptor contains 350 amino acid residues and is located in the chromosome 4q35.1 [496]. The MT_1_ receptor couples to G_αi/o_ protein which leads to the inhibition of cAMP formation, protein kinase A (PKA) activity and phosphorylation of CREB [497]. In addition, MT_1_ receptor couples to G_q/11_ protein increasing phosphatidylinositol turnover and intracellular calcium [497]. MT_1_ receptors can also increase the phosphorylation of the MAPK/ERK1/2 pathway, as well as potassium conductance through K_ir_ inwardly rectifying channels [498].

The MT_2_ receptor contains 362 amino acids residues and is located in the chromosome 11q21-q22 [499]. Similar to the MT_1_ receptor, the MT_2_ receptor is coupled to G_αi/o_ proteins inhibiting both forskolin-stimulated cAMP production and cGMP formation, activating protein kinase C (PKC) and decreasing calcium-dependent dopamine release in the retina [500].

The MT_3_ receptor has been characterized as a quinone reductase 2 enzyme (QR2, EC 1.10.99.2). The QR2-enzyme participates in the protection against oxidative stress by preventing electron transfer reactions of quinones [501].

Additionally, melatonin also exerts an effect on other targets, such as inhibiting the NMDA receptor in rat striatum [502], and enhancing central GABAergic transmission by modulating GABA receptor activity in the rat brain [503]. Other evidence suggests that melatonin may inhibit calcium influx resulting in inhibition of neuronal nitric oxide synthase (nNOS) activity, a decrease of NO production and an excitatory effect over NMDA receptors in the rat brain [504]. Besides, melatonin induces increases the affinity of the D2 receptors in the rat striatum [505]. All of this evidence suggests that melatonin could be a potent drug candidate for various neurological diseases such as epilepsy.

### Melatonin and Melatonin Receptors in Epilepsy

Melatonin is widely used for sleep disorders [506]. Besides, melatonin has antioxidant properties and is able to reduce neuronal excitability and protect the brain [493]. Based on these characteristics, different studies have started to assess a possible antiseizure effect by melatonin. However, the role of melatonin as an anticonvulsant hormone is due to the fact that it easily passes the BBB [507].

Much of the available literature points out to the anticonvulsant effects of melatonin in animal models. In vitro studies showed that melatonin depresses low-Mg^2+^ epileptiform neuronal activity through specific neocortical receptors, on human temporal neocortical slices [508]. In vivo, melatonin significantly raised the electroconvulsive threshold test in mice [509]; increased the after-discharge threshold and suppressed generalized seizures in the amygdala kindling model [510]; and increased the latency to epileptiform activity and decreased the frequency of spike and spike-wave activity, on penicillin-induced epileptiform activity in rats [511].

In addition, melatonin showed anticonvulsant effects when it was administered prior to the induction of hyperthermic febrile seizures in rats [512]. The subchronic treatment with melatonin increased the latency of pilocarpine-induced convulsions in rats and the number of [^3^H] GABA binding sites in hippocampal slices [513]. This suggests that melatonin actions might be due to positive modulation of GABAergic transmission [513]. Melatonin pretreatment before PTZ administration lowered the mortality rate, attenuated seizure severity and increased seizure latency in *guinea pigs* [514].

Pinealectomy (pineal gland removal), seemed to accelerate the amygdala kindling process by reducing the number of stimulations needed to reach fully kindled stage [515], suggesting that endogenous melatonin may act as a neuroprotective factor, as it was observed at pinealectomized epileptic rats, after SE induction in the pilocarpine model [516].

Although melatonin cotreatment did not significantly alter seizure activity, it improved the survival rate, attenuated KA-induced microglial activation and lipid peroxidation associated with hippocampal neurodegeneration, in melatonin cotreated rats [517,518]. In addition, melatonin increased the latency in the appearance of spontaneous recurrent seizures and decreased their frequency, while the hippocampal neuronal damage was reduced, in the KA-induced SE model [519]. However, long-term melatonin treatment after SE was unable to suppress the development of epileptogenesis [519]. These findings suggest that melatonin exerts a neuroprotective action against KA-induced excitotoxicity resulting from its role as a free radical scavenger, antioxidant, antiapoptotic or anti-inflammatory agent [517,518,519].

In the pilocarpine-induced SE model, animals treated with melatonin just before, during or after SE, exhibited an increased latency to SE, a survival rate of 100% after SE, a decreased number of seizures in the chronic period and hippocampal neuronal preservation [520]. Moreover, long-term melatonin administration after SE, reduced neuronal loss showing a region-specific pattern of neuronal protection along the rostro-caudal axis of the dorsal hippocampus [521]. These results confirm an important role for melatonin in the epileptogenic process as well as the control of seizures.

In relation to the mRNA expression levels of MT_1_ and MT_2_, they were increased in the rat hippocampus a few hours after pilocarpine-induced SE [522], but, during the silent phase, MT_1_ returned to normal levels while MT_2_ level was reduced [522]. This study suggests that pilocarpine-induced SE could modify melatonin-receptor proteins and mRNA expression levels in the hippocampus of rats.

With respect to melatoninergic ligands, the use of ramelteon (a MT_1_/MT_2_ agonist) showed anticonvulsant properties in the rapid kindling model, supporting melatonin-receptors as potential novel targets for anticonvulsant drug development [523]. VLB-01 (beprodone, a MT_3_/QR2 agonist), demonstrated an anticonvulsant profile in several animal models such as the MES test, PTZ, pilocarpine-induced seizures and PTZ-kindled model [524]. Additionally, VLB-01 has been tested in phase I and II clinical trials for the treatment of refractory focal seizures and focal onset seizures secondarily generalized, where it had a reduction on seizure frequency after 224 days, in comparison to placebo [524]. On the other hand, agomelatine (a MT_1_/MT_2_ agonist and 5HT_2C_ antagonist) had an anticonvulsant and neuroprotective action in chronic post-SE treatment [525].

Regarding human studies, it has been observed that in children with febrile seizures, there is a lower concentration of melatonin in both saliva and plasma [526,527]. Moreover, the plasma melatonin level showed an increase during seizures but a significantly lower level post-seizure [527]. Regarding patients with TLE, they showed low melatonin baseline levels, which increased following seizures [528]. In children with intractable epilepsy, diurnal melatonin plasma levels were decreased [529]. After children received oral melatonin for three months, 87% of patients reported an improvement in seizure frequency and seizure severity [529], a result that supports the use of melatonin in patients with intractable seizures and low melatonin levels given its anticonvulsant effect. In children with severe uncontrolled epilepsy (infantile spasms and Lennox–Gastaut syndrome) that were treated with melatonin plus AEDs for three months, a decrease in seizure frequency was reported [530].

Melatonin has shown to be an adjunctive treatment in the case of severe infantile myoclonic epilepsy [531]. Gupta et al. [532,533] investigated the effect of add-on melatonin on the blood levels of antioxidant enzymes (glutathione peroxidase and glutathione reductase), in epileptic children on valproate or carbamazepine monotherapy. They found an increase in the antioxidant activity of both enzymes, suggesting that melatonin exerts antioxidant activity in these patients [532,533]. Therefore, melatonin, as an adjunct, could exert a neuroprotective effect due to its antioxidant, antiexcitotoxic and free radical scavenging properties within the CNS.

However, oral administration of melatonin increases seizures in four out of five neurologically disabled young people with generalized tonic-clonic epilepsy [534]. Besides, the single case report of a 21-year-old woman with uncontrolled epilepsy under melatonin treatment, showed an increase in the epileptiform activity [535]. This evidence shows that melatonin promotes or worsens seizures in some epileptic patients.

Other authors reported that melatonin did not exacerbate seizures in epileptic children [536,537], but it was effective in treating sleep disorders [537]. However, a study in patients with severe epilepsy showed variable results as 27% of patients had an increased seizure rate, 63% of patients had a decrease and 10% of patients had no observable differences [538].

Melatonin can act as an anticonvulsant, an antioxidant and a neuroprotector in epilepsy. Melatonin and melatoninergic ligands may be suitable for use as add-on treatments with common AEDs. However, some studies suggest that melatonin worsens seizures or does not improve seizures in patients with epilepsy. Therefore, it is necessary to continue investigating how melatonin and melatonin-receptors act during the epileptogenesis process not only in preclinical models of epilepsy but also in drug-resistant epilepsy models.

## 12. Other Potential Therapeutic Targets to Consider

Some other therapeutic targets that have come to our attention are mentioned below, which might have a potential use in controlling epilepsy and drug-resistant epilepsy in the future.

### 12.1. G protein-Coupled Receptors

Other therapeutic targets are the G protein-coupled receptors (GPCRs), also known as the seven-transmembrane (7-TM) receptors, that comprise the largest super protein family of receptors that detect extracellular molecules and trigger signal transmission inside of the cell [539]. GPCRs might play some essential roles in the mediation of neuronal excitability via regulating Gαs and Gαi-controlled cAMP signaling, as well as Gαq-initiated Ca^2+^-sensitive pathways [539].

In this context, they have emerged as candidates for epileptogenic mediators owing to their essential roles in the regulation of ion channel functions, thereby altering neuronal excitability and setting the seizure threshold [539]. These receptors include prostanoid receptors, cannabinoid receptors, adenosine receptors, histamine receptors and metabotropic glutamate receptors (mGluRs). Understanding their role in seizure generation and development of epilepsy could help to develop new antiepileptic or antiepileptogenic drugs.

Among the prostanoid receptor subtypes, EP1, EP2 and FP are the most studied in acute seizures and epilepsy models [539,540,541,542,543,544,545,546,547,548,549,550,551,552]. The pharmacological inhibition or the genetic inactivation of the EP1 receptor demonstrated its contribution to increasing neuronal excitability and lowering the seizure threshold [540,542,543,548]. On the other hand, previous studies suggested that systemic pharmacologic inhibition of the EP2 receptor reduced brain inflammation and injury following pilocarpine-induced seizures in mice [545,546]. Later, it was demonstrated that pharmacological inhibition of the EP2 receptor (with TG4-155/TG6-10-1 antagonists) affords a broad spectrum of anti-inflammatory and neuroprotective effects in several rodent models of SE [547,553]. These findings support the notion of the EP2 receptor as an emerging target for adjunctive treatment, together with the current first-line anticonvulsants and glutamate receptor blockers, to prevent acute brain inflammation and damage following SE [547,553].

Concerning the endocannabinoid system, it consists of two currently known GPCRs: cannabinoid receptor type 1 (CB1) and type 2 (CB2) [554]. In contrast to the CB1 receptor that is generally considered as one of the most abundant GPCRs in the CNS, the CB2 receptor in normal conditions has very limited distribution in the brain [555,556]. Activation/inhibition of the cannabinoid receptors can modulate a wide range of intracellular and intercellular signaling activity, such as ion channels (i.e., potassium, sodium, and calcium channels), intracellular calcium ion concentrations, and inflammation [557]. In this way, CB1 and CB2 receptors can reduce neuronal activity and excitability of the brain, thereby generating interest and directing efforts into modulating these two inhibitory GPCRs as potential therapies for seizures or epilepsy [539,558,559]. It appears that the majority of studies about the CB1 receptor in various in vitro and in vivo models indicate that CB1 receptor activation causes anticonvulsant effects, while inhibiting CB1 receptors might aggravate convulsive seizures [560,561,562,563,564].

Since the CB2 receptor can be expressed by activated brain microglia, this receptor has emerged as an appealing anti-inflammatory target for brain conditions. Recently, one study suggests that targeting the CB2 receptor with the selective inverse agonist SMM-189, might represent an adjunctive therapeutic strategy for reducing brain inflammation and damage following prolonged seizures, together with first-line anticonvulsant agents and glutamate receptor blockers [565].

In relation to endocannabinoid receptors and drug-resistant epilepsy, a study revealed reduced mRNA and protein expression levels of CB1 receptors in the hippocampus of patients with drug-resistant TLE, especially in glutamatergic axon terminals [566]. Recently, it was demonstrated that drug-resistant mesial TLE is associated with a higher CB1 receptor-induced Gi/o protein activation level, as well as an increased tissue content of specific endocannabinoids in the hippocampus and the temporal neocortex [567].

However, although cannabinoids show promise as an anticonvulsant and anti-inflammatory treatment, their promiscuous binding affinity for numerous receptors makes the task of identifying their beneficial and detrimental mechanisms of action challenging [559].

### 12.2. BDNF/TrkB Signaling

Neurotrophin brain-derived neurotrophic factor (BDNF) is the most widely studied neurotrophin in the mammalian nervous system [568]. Via its tropomyosin-related kinase receptor B (TrkB), this neurotrophin regulates a variety of physiological processes such as learning, memory and reward [568,569]. BDNF/TrkB signaling is thought to play a critical role in epilepsy [570,571].

BDNF expression increases following epileptic seizures in experimental animals and patients with TLE [572,573,574,575]. Intracerebroventricular infusion of antibodies that recognize TrkB blocked the kindling development in rats [576]. On the other hand, the increased TrkB signaling is related to seizure induction in a mouse kindling model [577,578].

The fact that the downstream effector phosphoinositide-specific phospholipase C-γ1 (PLC-γ1) seems essential to BDNF/TrkB-mediated epileptogenesis in the kindling model [579] also points to BDNF/TrkB/PLCγ1 signaling as a possible molecular mechanism by which SE induces TLE [580,581] providing a new therapeutic target for possible epilepsy prevention.

Recent findings suggest that COX-2 via PGE2/EP2 signaling regulates the hippocampal BDNF/TrkB pathway following prolonged seizures. Thus, EP2 inhibition by brain-permeable antagonists such as TG6-10-1 might provide a novel strategy to suppress the abnormal TrkB activity, an event that can trigger epileptogenesis and consequently generate epilepsy [582].

Taken together, these studies provide evidence of the contribution of BDNF/TrkB signaling to epileptogenesis, the process by which a normal brain becomes epileptic. By selectively disrupting pathways downstream or upstream it could be possible to regulate BDNF/TrkB signaling, which would hopefully result in the treatment not only of epilepsy but also of drug-resistant epilepsy, where the use of matrix encapsulated cell lines secreting human BDNF provides an interesting option for patients with drug-resistant TLE [583].

### 12.3. Pannexins

Pannexins, a family of membrane proteins, are nonselective, large-pore channels mediating extracellular exchange of neuroactive molecules [584,585,586]. Three proteins coded by pannexin genes have been identified: pannexin-1 and pannexin-2 are abundantly found in the central nervous system (CNS), while pannexin-3 is not [587]. In the CNS, pannexin-1 channels are expressed in both neurons and astroglia [587,588,589,590]; they mediate the release of adenosine 5′-triphosphate (ATP) and the excitatory neurotransmitter glutamate [591]; and they have a vital role in modulating cellular hyperexcitability [584,585,586].

Recent data suggest that these channels are activated under pathological conditions such as epilepsy and regulate neuronal excitability [586,592,593,594,595]. Pannexin-1 expression seems raised in animal seizure models and in resected human epileptic brain tissue, suggesting relevance to epilepsy [586,596,597,598,599]. Recently, it was reported that pannexin-1 channel activation promotes seizure generation and maintenance through adenosine triphosphate signaling via purinergic 2 receptors [600]. Genetic or pharmacological targeting of pannexin-1 channels have anticonvulsant effects in epilepsy models [595,600]. These results suggest that pannexin-1 channel inhibition might represent an alternative therapeutic strategy for treating not only epilepsy but also drug-resistant epilepsy.

## 13. Conclusions and Future Perspectives

For the development of new treatment tools, it is necessary to continue exploring key targets associated with the progression of chronic epilepsy. We provide an overview of promising research directions in drug therapy not only for seizures or epilepsy but also for drug-resistant epilepsy.

Starting with molecular pathways such as mTOR and Wnt/β-catenin pathways, which are often disrupted following seizures and in chronic epilepsy, they could be considered as favorable antiepileptogenic targets for future drug therapies, as their inhibition has beneficial effects as protecting against neuronal damage and death. With respect to zinc supplementation as a therapeutic strategy, it is necessary to continue investigating this line of research, as there exist different sources of zinc and because it modulates a variety of ion channels and receptors, resulting in pro- or anticonvulsant effects besides neuronal neurotoxicity.

On the other hand, carbonic anhydrase inhibitors have shown anticonvulsant effects, but it is still necessary to identify and evaluate new specific inhibitors such as carbonic anhydrase-VII inhibitors, as anticonvulsant or antiepileptogenic drugs. For erythropoietin and erythropoietin-derived mimetic peptides, it has been reported they have neuroprotective or anti-inflammatory effects. Copine 6 (neuronal-copine) expression is significantly increased in the epileptic brain where it seems to be involved in controlling different signaling pathways and synaptic plasticity. However, only recently has it begun to be elucidated its role in epilepsy. The complement system (complex innate immune system) seems to contribute to seizures as well as neurodegeneration after its excessive activation. Thus, inhibition of key complement proteins might avoid seizure generation and epileptogenesis process. The inhibition of C5a complement receptor 1 (C5ar1) or the use of an anti-C5 antibody (eculizumab) has not yet been tested in epilepsy or drug-resistant epilepsy, but both targets are promising as epileptic therapeutics tools.

Antagonism of TRPV1 channels, peptidomimetics, non-peptide agonists or positive allosteric modulators of galanin, have also displayed anticonvulsant, antiepileptogenic and neuroprotective actions. Melatonin and melatoninergic ligands may exert neuroprotective effects due to its antioxidant, antiexcitotoxic and free radical scavenging properties besides their anticonvulsant effects, suggesting their application as an adjunct therapy.

All these evidences expose promising therapeutic approaches for the development of pharmacological therapies to control epileptic seizures, to prevent the onset and progression of chronic epilepsy, and in their potential use in drug-resistant epilepsy as well. However, further research is still necessary to evaluate how these therapeutic interventions can be applied safely without causing undesirable side effects, especially in combination with already commonly prescribed AEDs, with the end goal of translating preclinical and clinical findings and research to the development of new and safe therapeutic drugs.

Finally, another point worth considering and researching is the simultaneous modulation of two or more of these key therapeutic approaches as a promising avenue to prevent the development of chronic epilepsy and drug-resistant epilepsy, without losing sight of the broader possible effects on other functions mediated by these therapeutic targets, including those that are critical for the regulation of many physiological functions and normal cellular processes.

## Abbreviations

TLE	temporal lobe epilepsy
AEDs	antiepileptic drugs
KA	kainic acid
SE	status epilepticus
PTZ	Pentylenetetrazole
BBB	blood–brain barrier
CNS	central nervous system
